# Association of intraplacental oxygenation patterns on dual‐contrast MRI with placental abnormality and fetal brain oxygenation

**DOI:** 10.1002/uog.24959

**Published:** 2023-01-12

**Authors:** Z. Sun, W. Wu, P. Zhao, Q. Wang, P. K. Woodard, D. M. Nelson, A. Odibo, A. Cahill, Y. Wang

**Affiliations:** ^1^ Department of Biomedical Engineering Washington University in St Louis St Louis MO USA; ^2^ Department of Obstetrics and Gynecology Washington University School of Medicine, Washington University in St Louis St Louis MO USA; ^3^ Mallinckrodt Institute of Radiology Washington University School of Medicine, Washington University in St Louis St Louis MO USA; ^4^ Department of Women's Health University of Texas at Austin, Dell Medical School Austin TX USA; ^5^ Department of Electrical & Systems Engineering Washington University in St Louis St Louis MO USA

**Keywords:** diffusion MRI, fetal brain oxygenation, intervillous space oxygenation, intraplacental segmentation, T2* MRI, tissue‐specific

## Abstract

**Objectives:**

Most human *in‐vivo* placental imaging techniques are unable to distinguish and characterize various placental compartments, such as the intervillous space (IVS), placental vessels (PV) and placental tissue (PT), limiting their specificity. We describe a method that employs T2* and diffusion‐weighted magnetic resonance imaging (MRI) data to differentiate automatically placental compartments, quantify their oxygenation properties and identify placental lesions (PL) *in vivo*. We also investigate the association between placental oxygenation patterns and fetal brain oxygenation.

**Methods:**

This was a prospective study conducted between 2018 and 2021 in which dual‐contrast clinical MRI data (T2* and diffusion‐weighted MRI) were acquired from patients between 20 and 38 weeks' gestation. We trained a fuzzy clustering method to analyze T2* and diffusion‐weighted MRI data and assign placental voxels to one of four clusters, based on their distinct imaging domain features. The new method divided automatically the placenta into IVS, PV, PT and PL compartments and characterized their oxygenation changes throughout pregnancy.

**Results:**

A total of 27 patients were recruited, of whom five developed pregnancy complications. Total placental oxygenation level and T2* did not demonstrate a statistically significant temporal correlation with gestational age (GA) (*R* ^2^ = 0.060, *P* = 0.27). In contrast, the oxygenation level reflected by T2* values in the placental IVS (*R* ^2^ = 0.51, *P* = 0.0002) and PV (*R* ^2^ = 0.76, *P* = 1.1 × 10^−7^) decreased significantly with advancing GA. Oxygenation levels in the PT did not show any temporal change during pregnancy (*R* ^2^ = 0.00044, *P* = 0.93). A strong spatial‐dependent correlation between PV oxygenation level and GA was observed. The strongest negative correlation between PV oxygenation and GA (*R* ^2^ = 0.73, *P* = 4.5 × 10^−7^) was found at the fetal‐vessel‐dominated region close to the chorionic plate. The location and extent of the placental abnormality were automatically delineated and quantified in the five women with clinically confirmed placental pathology. Compared to the averaged total placental oxygenation, placental IVS oxygenation level best reflected fetal brain oxygenation level during fetal development.

**Conclusion:**

Based on clinically feasible dual‐MRI, our method enables accurate spatiotemporal quantification of placental compartment and fetal brain oxygenation across different GAs. This information should improve our knowledge of human placenta development and its relationship with normal and abnormal pregnancy. © 2022 The Authors. *Ultrasound in Obstetrics & Gynecology* published by John Wiley & Sons Ltd on behalf of International Society of Ultrasound in Obstetrics and Gynecology.

**CONTRIBUTION**

*What are the novel findings of this work?*

Our clustering method employing T2* and diffusion‐weighted magnetic resonance imaging (MRI) data enabled automatic segmentation of the placenta into intervillous space, placental vessels, placental tissue and placental lesion. It also allowed spatiotemporal characterization of the placental compartments. When compared with averaged total placental oxygenation, placental intervillous space oxygenation has a stronger association with fetal brain oxygenation during fetal development.

*What are the clinical implications of this work?*

This work provides a basis for using dual‐contrast MRI for evaluating placental and fetal brain oxygenation in pregnancy complicated by placental malperfusion.

## INTRODUCTION

The placenta supports fetal development by supplying oxygen, cytokines, growth factors and nutrients and removing carbon dioxide and waste products^1^. In humans, placental abnormalities can lead to pregnancy complications, such as pre‐eclampsia (PE), intrauterine growth restriction and preterm birth (PTB), all of which harm the developing fetus. Furthermore, the development of the central nervous system has been shown to be related closely to placental function^2^.

Our understanding of placental abnormalities comes mainly from analyzing the placenta after delivery^3^, by which time the placental structure and functional properties may have changed substantially^4^, ^5^, ^6^. To examine the human placenta *in vivo*, researchers have used ultrasound and magnetic resonance imaging (MRI). The MRI method involving T2* quantification allows assessment of the distribution of deoxyhemoglobin. Deoxyhemoglobin yields lower T2* values and thus reflects blood and oxygen supply to the organ^7^. Although ultrasound and T2* mapping are useful for imaging the human placenta, these methods yield measures that are averaged over the entire placenta or a region of interest containing various placental compartments and cell types^8^, ^9^, ^10^, ^11^, ^12^, ^13^, resulting in reduced imaging specificity and contradictory findings^14^, ^15^. Considering the heterogeneous composition of the human placenta, quantifying oxygenation levels separately for individual placental compartments holds great promise to improve the characterization of human placental development and disease mechanism.

We describe a new method of imaging the placenta, which combines clinical T2* imaging and diffusion‐weighted MRI to allow automatic identification of four placental compartments: intervillous space (IVS), placental vessels (PV), placental tissue (PT) and placental lesions (PL). Our hypothesis was that data from individual compartments identified using fuzzy clustering machine‐learning methodology would be different and more specific compared with T2* data that are averaged over the entire placenta. We also aimed to assess the association between T2* measurements of individual compartments *vs* the entire placenta and fetal brain oxygenation.

## METHODS

### Study design

This was a prospective study conducted between 2018 and 2021. The study was approved by the institutional review board of Washington University in St Louis, St Louis, MO, USA (IRB number: 201707152). Patients with a singleton pregnancy at low risk of PTB (i.e. normal cervical length and no history of spontaneous PTB), receiving obstetric care and delivering at our institute (Department of Obstetrics and Gynecology, Washington University School of Medicine, Washington University in St Louis, St Louis, MO, USA) were recruited. The risk of PTB was estimated based on patient medical and pregnancy history. Exclusion criteria were multiple gestation, nulliparity, contraindication to progesterone therapy, major fetal anomaly at recruitment and contraindication to MRI.

The study was explained to the patients, and those willing to participate provided written informed consent. MRI was performed using a 3‐Tesla Siemens Vida scanner (Siemens, Erlangen, Germany). MRI was scheduled 1 month after patient recruitment and was performed without additional oxygen administration. The methodology of T2* and diffusion‐weighted MRI data analysis is described in Appendix S1.

### Fuzzy clustering model and expectation maximization

We trained a fuzzy clustering method based on the Gaussian mixture model to analyze both T2* and diffusion‐weighted MRI data and assign placental voxels to one of four clusters based on their distinct imaging domain features. This unsupervised fuzzy clustering method computes the probability of each voxel belonging to a certain distribution. Any voxel with a posterior probability lower than 0.05 for all clusters was regarded as an outlier and assigned to an additional cluster representing abnormal voxels. The mathematical details of the fuzzy clustering model are provided in Appendix S2 (Figures S1 and S2; Table S1).

### Method validation

To assess the reproducibility of the method, cross‐validation was conducted using a random subset of all collected data (Appendix S2). To validate further the accuracy of the method, an experienced radiologist (P.K.W.) and obstetrician (D.M.N.) manually selected and labeled regions containing IVS, PV and PT from five patients. The T2* and diffusion parameters from the manually selected imaging voxels were extracted and compared with the automatic clustering results (Figure S3).

### Fetal brain analysis

For each case, fetal brain segmentation was performed. Fetal brain volume and average T2* in healthy and complicated pregnancies were extracted, and linear regression was used to analyze the relationship between fetal brain and placental parameters.

## RESULTS

### Study population

A total of 27 patients were recruited, of whom five developed pregnancy complications according to clinical or pathological examination (Figure S4). The specific pregnancy complications seen in the placenta of the five women included fibrin deposition, thrombus formation, placental edema, chorionitis, thrombohematomas and fetal inflammatory response (Table 1). MRI parameters of complicated pregnancies are presented in Table 2.

**Table 1:** Demographic and clinical characteristics of five patients with complicated pregnancy

Characteristic	Case 1	Case 2	Case 3	Case 4	Case 5
Maternal age (years)	31	22	30	27	26
BMI (kg/m^2^)	21.5	20.9	35.7	39	39
Pregnancy history*	G7P1233	G3P2002	G2P0101	G4P1112	G3P1102
Medical history	No medical history noted	Attention deficit disorder; bipolar disorder	Severe chronic hypertension; PE; HELLP; depression (Zoloft)	Oligohydramnios; increased LFT; PE in previous and current pregnancies; ARDS; cholecystitis; acute kidney injury; right UPJ obstruction; GBS positive	Syphilis (treated); PE; placental abruption; anemia
GA at delivery (weeks)	36 + 6	39 + 2	34 + 5 with Apgar score of 6 at 1 min and 8 at 5 min	35 + 5	37 + 0 via CS
Placental findings†	Subchorionic fibrin; intervillous thrombus; yellow lesion in center of placenta, measuring 1 cm in maximum diameter	Unremarkable three‐vessel cord and fetal membrane; mature chorion with focal villous edema	Fetal membranes with pigment‐laden macrophages; intervillous thrombohematomas	Acute chorionitis; trivascular cord with umbilical arteritis (mild/focal fetal inflammation response)	Unremarkable fetal membranes and trivascular cord
Pregnancy outcome	Live birth	Live birth	Live birth	Live birth	Live birth
Fetal gender	Male	Female	Male	Male	Female
BW (g)	3380	2950	2650	3240	3160
Neonatal length (cm)	51	50	50	50	51.5
Neonatal HC (cm)	35	35	35	35	35

* Pregnancy history is presented as follows: G (gravidity) P (term birth/preterm birth/abortion or miscarriage/live birth).

† Based on pathology report.

ARDS, acute respiratory distress syndrome; BMI, body mass index; BW, birth weight; CS, Cesarean section; GA, gestational age; GBS, Group‐B streptococcus; HC, head circumference; LFT, liver function tests; PE, pre‐eclampsia; UPJ, ureteropelvic junction.

**Table 2:** Mean values of magnetic resonance imaging parameters in placental lesion cluster of five patients with complicated pregnancy

Parameter	Case 1	Case 2	Case 3	Case 4	Case 5
ADC (mm^2^/s)	1.43 ± 0.28	2.29 ± 0.34	2.12 ± 0.31	1.26 ± 0.12	2.98 ± 0.17
FA	0.27 ± 0.06	0.21 ± 0.05	0.22 ± 0.04	0.49 ± 0.07	0.43 ± 0.06
AD (mm^2^/s)	1.67 ± 0.80	2.43 ± 0.93	2.41 ± 0.67	2.01 ± 0.51	3.93 ± 0.61
RD (mm^2^/s)	1.33 ± 0.26	2.22 ± 0.22	2.35 ± 0.26	1.01 ± 0.15	1.95 ± 0.17
T2* (ms)	44.2 ± 9.9	60.6 ± 9.4	61.4 ± 14.0	32.9 ± 5.4	33.6 ± 6.7

Data are given as mean ± SD.

AD, axial diffusivity; ADC, apparent diffusion coefficient; FA, fractional anisotropy; RD, radial diffusivity.

### Identification of placental compartments using T2* and diffusion‐weighted MRI

**Figure 1:**
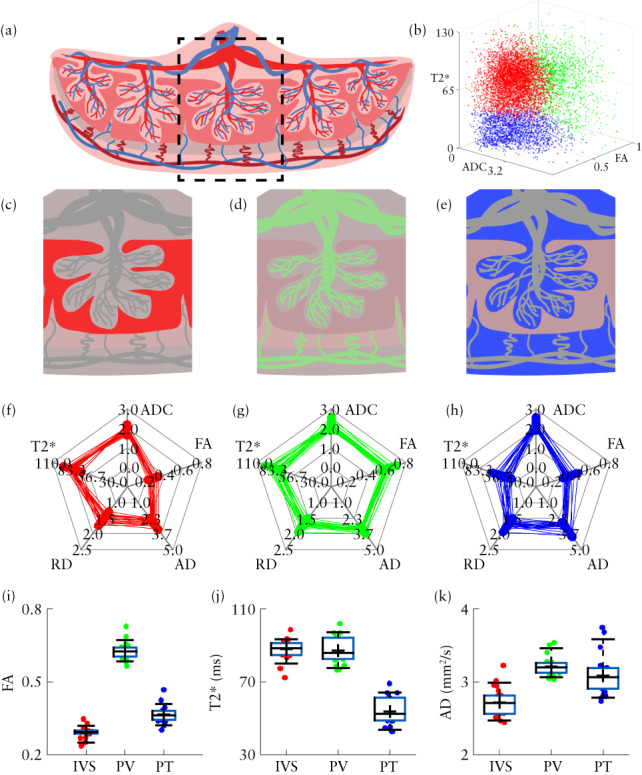
(a) Cross‐sectional schematic diagram of placenta showing five cotyledons. (b) Three‐dimensional graph showing multivariate mixture distribution. (c–e) Schematic diagrams revealing three compartments of the cotyledon outlined with dashes in (a): intervillous space (IVS) (c), maternal and fetal placental vessels (PV) (d) and placental tissue (PT) (e). (f–h) Radar plots displaying magnetic resonance imaging parameters of 22 healthy pregnancies in each cluster. (i–k) Boxplots showing mean fractional anisotropy (FA) (i), T2* (j) and axial diffusivity (AD) (k) values of the three clusters. Each circle represents one patient, box is interquartile range, horizontal line within box is median, whiskers are 5^th^ and 95^th^ percentiles and + is mean. All analyses were performed on three‐dimensional data. ADC, apparent diffusion coefficient; RD, radial diffusivity.

**Figure 2:**
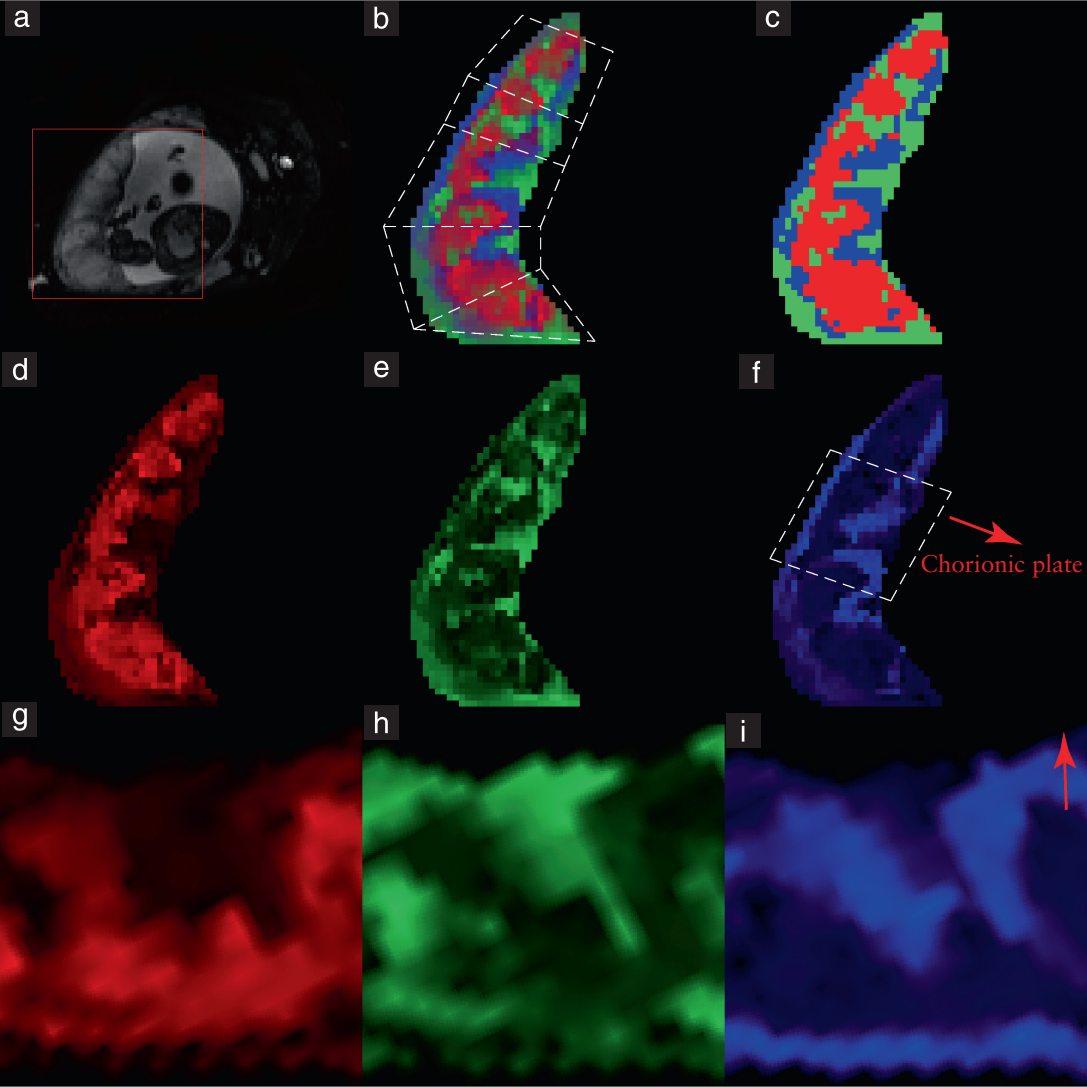
Placental segmentation in an uncomplicated pregnancy. (a) B0 image. (b) Map in which each voxel is assigned a color representing the probability with which that voxel matches each cluster. Dashed lines indicate five proposed cotyledons, each with intervillous space, placental vessels and placental tissue in the anticipated locations. (c) Map in which each voxel is assigned to a single cluster according to a threshold value. (d–f) Maps showing the individual color maps from (b). (g–i) High‐magnification images of the region in (d–f) corresponding to the area outlined with dashed white lines in (f) (arrows indicate orientation).

We trained and validated a fuzzy clustering model using T2* mapping and diffusion‐weighted MRI data from 22 uncomplicated pregnancies to identify three clusters corresponding to the physiological placental compartments IVS, PV and PT (Figure 1). Each cluster was characterized by a unique shape of the radar map of key imaging parameters (Figure 1f–h); the values of imaging parameters in each cluster are presented in Figure 1i–k. An example of placental segmentation from one of the cases is shown in Figure 2. We refer to the three clusters as C1‐IVS, C2‐PV and C3‐PT. Further details on the specific regions of the placenta from which signals were received are provided in Appendix S3.

**Figure 3:**
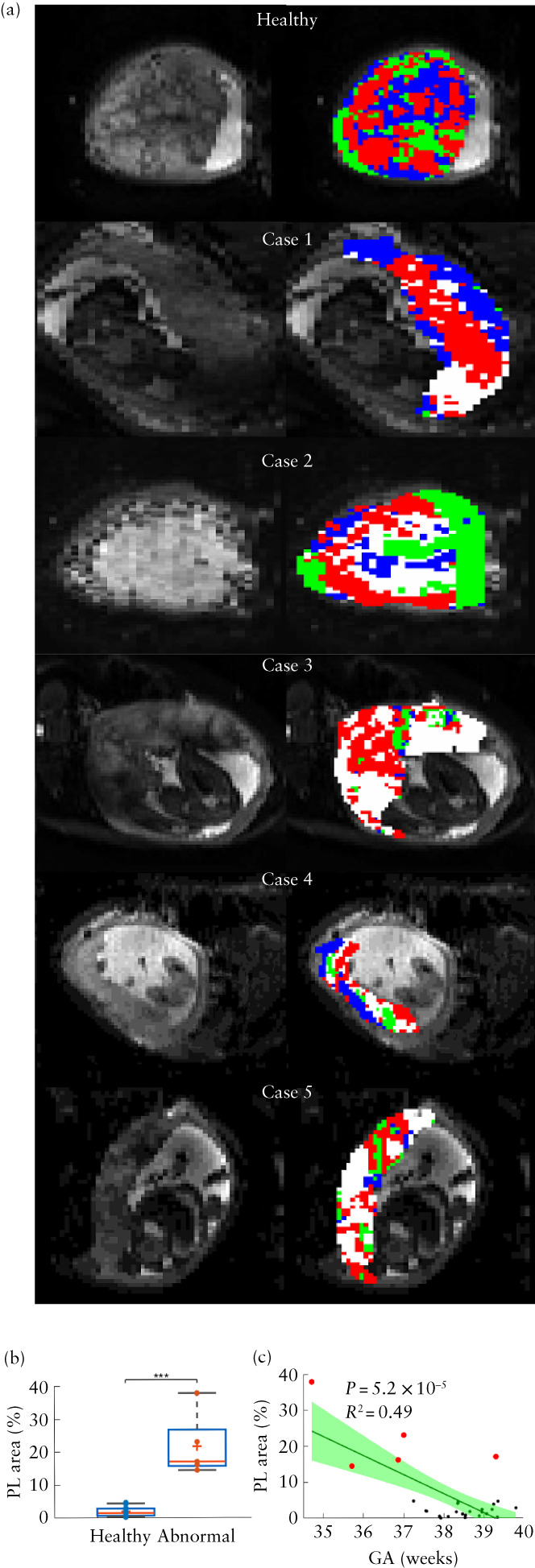
(a) B0 images of the placentas (left column) and the same images overlaid with a map in which each voxel is assigned to a single cluster (right column) obtained in one healthy patient and five patients with a complicated pregnancy. Red indicates intervillous space, green indicates placental vessels, blue indicates placental tissue and white indicates abnormal voxels (placental lesion (PL)). All patients underwent magnetic resonance imaging at 32 weeks' gestation, except Case 5, which underwent magnetic resonance imaging at 20 weeks' gestation. (b) Boxplot demonstrating PL area as a proportion of the total placental area in healthy and complicated pregnancy groups (***P < 0.001). Box is interquartile range, horizontal line within box is median, + is mean and whiskers are range. (c) Linear regression between PL area proportion and gestational age (GA) at delivery among abnormal (red circle) and healthy (black circle) cases.

On clustering analysis, in the placentas from healthy pregnancies, fewer than 6% of voxels did not meet the threshold to be assigned to any of the three compartments. In the placentas from the five complicated pregnancies, 14–38% of voxels could not be assigned to any of the three compartments. These voxels were denoted as abnormal and labeled as C4‐PL (Figure 3a). C4‐PL area, expressed as a proportion of the total placental area, of healthy and complicated pregnancies is presented in Figure 3b. Additionally, a statistically significant linear correlation was observed between C4‐PL area and gestational age (GA) at delivery (Figure 3c).

### Spatiotemporal T2* MRI changes in placenta with gestation

**Figure 4:**
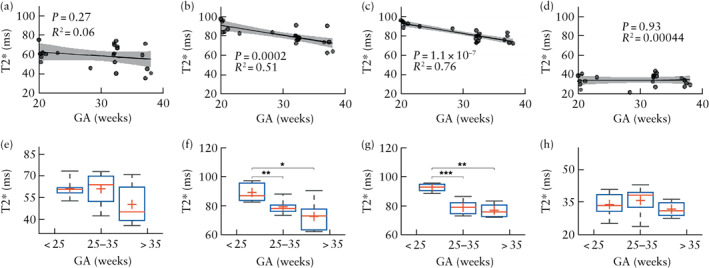
Scatterplots (a–d) and boxplots (e–h) showing T2* values of the total placenta (a,e) and individual placental compartments, including intervillous space (b,f), placental vessels (c,g) and placental tissue (d,h), according to gestational age (GA). In scatterplots, each circle represents the average T2* value from a single patient with a healthy pregnancy, and gray shaded regions denote 95% CI on linear regression. In boxplots, sample size was as follows for each GA category: *n* = 7 for < 25 weeks, *n* = 10 for 25–35 weeks and *n* = 5 for > 35 weeks. Mann–Whitney *U*‐test was used to compare groups: *P < 0.05; **P < 0.01; ***P < 0.005.

To determine whether oxygenation differences between placental compartments can be detected across gestation, we computed and plotted the T2* values of each compartment and the entire placenta for 22 healthy pregnancies against GA (Figure 4). When measured for the entire placenta, T2* was lower at later GAs; however, the difference was not significant on linear regression (Figure 4a) or Mann–Whitney *U*‐test (Figure 4e). Strikingly, the T2* values of the C1‐IVS and C2‐PV were significantly lower in the placentas examined at later GAs (Figure 4b,c,f,g).

**Figure 5:**
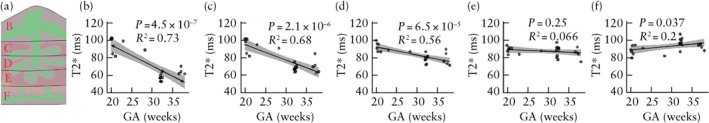
(a) Schematic diagram indicating the five layers of the placental vessels (PV) compartment in our analysis, with layer B on the chorionic (fetal) side and layer F on the basal (maternal) side. (b–f) Scatterplots demonstrating T2* values of the five placental layers, including the chorionic (b), near‐chorionic (c), middle (d), near‐basal (e) and basal (f) layers, according to gestational age (GA). Each circle represents the average T2* value of the PV layer in individual patients with a healthy pregnancy. Gray regions denote 95% CI on linear regression.

Human placental anatomy suggests that, within the PV compartment, there are fetal‐derived vessels on the chorionic side of the placenta and maternal‐derived vessels on the basal side. To study the differences of these vessels on imaging, we divided C2‐PV into five layers with respect to their depth relative to the chorionic and basal sides. The mean T2* values of each layer of the C2‐PV were plotted according to GA (Figure 5). Linear regression of each plot revealed that T2* decreased significantly across gestation in the layer closest to the chorion (Figure 5b) and the next two layers (Figure 5c,d), with the steepest slope across gestation in the layer closest to the chorion. However, T2* values did not differ significantly with GA in the near‐basal layer (Figure 5e) and had a positive association with GA in the layer closest to the maternal plate (Figure 5f). Similar layer‐wise analysis in the C1‐IVS, C3‐PT and entire placenta showed that the relationship between T2* values and GA was similar in each layer (Figures S5 and S6).

### Association between placental and fetal brain oxygenation

**Figure 6:**
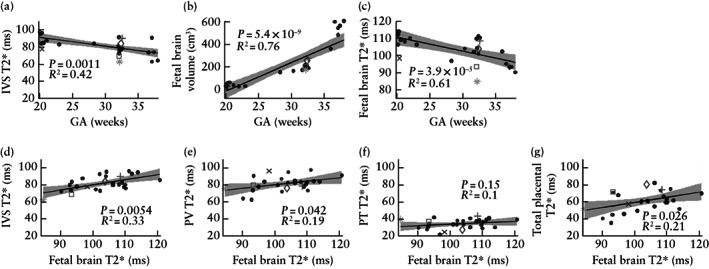
Scatterplots demonstrating the relationship on linear regression between intervillous space (IVS) T2* values and gestational age (GA) (a), fetal brain volume and GA (b), fetal brain T2* values and GA (c), IVS T2* and fetal brain T2* values (d), placental vessels (PV) T2* and fetal brain T2* values (e), placental tissue (PT) T2* and fetal brain T2* values (f) and total placental T2* and fetal brain T2* values (g). Gray regions denote 95% CI. 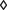, Case 1; 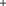, Case 2; 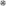, Case 3; 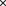, Case 4; 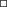, Case 5; 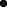, healthy.

We assessed whether oxygenation measurements of placental compartments reflected brain oxygenation during fetal development (Figure 6). As expected, we found that fetal brain volume increased and fetal brain T2* decreased across gestation, reflecting fetal brain growth and increased oxygen consumption (Figure 6b,c). Moreover, fetal brain T2* values of four complicated pregnancies (Cases 2, 3, 4 and 5) were outside of the 95% CI (Figure 6c). On linear regression between placental and fetal brain T2* values, fetal brain T2* values demonstrated the strongest correlation with IVS T2* values (Figure 6d).

## DISCUSSION

In this study, we present a fuzzy clustering method that can employ clinical MRI data (T2* and diffusion‐weighted MRI) for automatic dissection of the placenta into placental compartments IVS, PV and PT, and identification of PL. Despite the previous findings of placental inhomogeneity^16^, ^17^, most of the previously published placental imaging methods quantified imaging biomarkers across the entire placenta. This has generated conflicting evidence regarding the pattern of changes in placental oxygenation with advancing GA^15^, ^18^, ^19^, ^20^, ^21^. In contrast, our method enabled automatic, specific spatiotemporal characterization of oxygenation distribution in intraplacental compartments during human pregnancy.

This study demonstrated a strong negative correlation between oxygenation levels in C1‐IVS and C2‐PV (but not in C3‐PT) and GA, which is consistent with the increasing metabolic demand of the placenta and increasing oxygenation transfer to fetal circulation^22^. In addition, the T2* gradient in normal pregnancies matched well with the placental oxygenation pattern required to support gas diffusion^22^.

This study found that the placentas from healthy pregnancies had fewer C4‐PL voxels (< 6%) compared with the placentas from complicated pregnancies, which had up to 38% of C4‐PL voxels. In the five patients with a complicated pregnancy, reduced T2* values in the C4‐PL were observed, suggesting reduced oxygen levels and compromised placental function in this region.

In the placentas with a severe lesion (Cases 1 and 4), we found reduced apparent diffusion coefficient (ADC), axial diffusivity, radial diffusivity (RD) and T2* values in the C4‐PL region, which is in agreement with the reported local inflammation and limited blood supply associated with severe placental lesion^23^. In addition, the decreased placental T2* and ADC in Cases 1 and 4 reflect limited oxygen supply associated with placental insufficiency, which is in agreement with the results reported by Bonel *et al*.^23^. In the placenta with local edema (Case 2), diffusion MRI findings included increased RD and decreased fractional anisotropy^24^, ^25^. Both PTB cases (Cases 3 and 4) had inflammation in fetal compartments, which may be explained by the strong association between fetal inflammation and imminent preterm labor^26^. Given that placental immune cell infiltration is considered to be the major source of fetal inflammation^27^, we hypothesize that the C4‐PL area may serve as a potential marker for PTB prediction, as the preliminary analysis showed a significant association between PL area and GA at delivery (Figure 3c). Moreover, Case 3 had the highest C4‐PL area of all five patients; the dysfunction seen in over one‐third of the patient's placenta is likely to be the consequence of severe chronic hypertension. In pregnancy with maternal anemia, we found increased extracellular diffusion (Case 5). This is related possibly to the abnormally increased total volume of the placenta (1216 cm^3^ at 32 weeks' gestation, above the 97^th^ percentile), which is observed typically in pregnancy with maternal anemia.

The significant correlation between fetal brain T2* and placental C1‐IVS T2* values suggests that the maternal blood oxygenation level in IVS, estimated by our method, can serve as an accurate and sensitive placental biomarker of fetal brain oxygenation in both normal and abnormal pregnancy. Such correlation between placental function and fetal brain condition has been reported by a study of Luo *et al*.^28^, in which greater placental time to plateau was associated with a smaller fetal brain. All three patients with PE (Cases 3, 4 and 5) had lower placental and fetal brain T2* compared with healthy subjects at the same GA, suggesting lower placental and fetal brain oxygenation. This finding of fetal hypoxia in PE matches the findings of previous research^29^.

The major limitation of this study is the small sample size and the heterogeneity of pregnancy disorders in the complicated group, which limited our ability to draw specific conclusions regarding T2* changes in each type of pregnancy complication, such as PE, PTB and fetal growth restriction. However, this study provides a solid basis for more extensive studies in the future to validate further our technique and findings.

In conclusion, although some MRI studies have identified placental differences between normal and abnormal pregnancies^15^, ^30^, ^31^, they could not generate a quantitative index reflecting the severity of placental pathology. In contrast, our automated clustering system provides the percentage of abnormal placental voxels. Therefore, it may serve as an objective and quantitative method to assess human placental health *in vivo* during pregnancy. Future research may focus on employing this clustering technique to examine a larger cohort, assess each pregnant woman at multiple timepoints throughout pregnancy to obtain longitudinal data and investigate the changes in placental structure and function across gestation.

## Supporting information


**Appendix S1** Magnetic resonance image processing methodology
**Appendix S2** Detailed description of the fuzzy clustering method based on the Gaussian mixture model and cross‐validation
**Appendix S3** Detailed description of the three identified clusters
**Table S1** Repeated training process using different sample volume
**Figure S1** Repeated training process using different sample volume. (a) Mean values of randomly selected 10 000 voxels from 22 patients (repeated 10 times). (b) Mean values of randomly selected 10 000 voxels from six patients (repeated 10 times).
**Figure S2** (a–c) 95% CI of three‐dimensional multivariable distribution. Red indicates intervillous space cluster, green indicates placental vessels cluster and blue indicates placental tissue cluster. (d–f) Planes showing the position of corresponding two‐dimensional projections: 95% CI on apparent diffusion coefficient (ADC)‐fractional anisotropy (FA) plane (d), 95% CI on ADC‐T2* plane (e), 95% CI on FA‐T2* plane (f).
**Figure S3** Validation by manually selected voxels.
**Figure S4** Flowchart summarizing selection of study subjects.
**Figure S5** Spatial distribution of placental compartments and oxygenation levels.
**Figure S6** Full spatiotemporal analysis.

## Data Availability

The data that support the findings of this study are available from the corresponding author upon reasonable request.
