# Supplementary material for: Association of intraplacental oxygenation patterns on dual‐contrast MRI with placental abnormality and fetal brain oxygenation
Source: Ultrasound Obstet Gynecol. 2023 Jan 12;61(2):215–23. doi: 10.1002/uog.24959 (PMC9708928; doi:10.1002/uog.24959)
Supplement: Supplementary file 1 — Appendix S1 Magnetic resonance image processing methodology Appendix S2 Detailed description of the fuzzy clustering method based on the Gaussian mixture model and cross‐validation Appendix S3 Detailed description of the three identified clusters Table S1 Repeated training process using different sample volume Figure S1 Repeated training process using different sample volume. (a) Mean values of randomly selected 10 000 voxels from 22 patients (repeated 10 times). (b) Mean values of randomly selected 10 000 voxels from six patients (repeated 10 times). Figure S2 (a–c) 95% CI of three‐dimensional multivariable distribution. Red indicates intervillous space cluster, green indicates placental vessels cluster and blue indicates placental tissue cluster. (d–f) Planes showing the position of corresponding two‐dimensional projections: 95% CI on apparent diffusion coefficient (ADC)‐fractional anisotropy (FA) plane (d), 95% CI on ADC‐T2* plane (e), 95% CI on FA‐T2* plane (f). Figure S3 Validation by manually selected voxels. Figure S4 Flowchart summarizing selection of study subjects. Figure S5 Spatial distribution of placental compartments and oxygenation levels. Figure S6 Full spatiotemporal analysis. [file UOG-61-215-s001.docx]

**Appendix S1** Magnetic resonance image processing methodology

**MRI Scan Parameters**

Diffusion MR images and multi-echo T2* MR images were acquired from 22 patients with uncomplicated singleton pregnancies and 5 patients with complicated pregnancies. Details, including diagnoses and pathological assessment of the placentas from complicated pregnancies, are provided in **Table 1.** Each patient underwent one MRI scan between 20 weeks' and 38 weeks' gestation. For all MRI scans, patients laid in the left lateral position, and T2* and diffusion scans covered the same fields of view. Within each scan, two sections of T2* MR imaging were acquired by multi-echo T2* sequence. The echo trains of the first and second sections were [3 ms, 5.11 ms, 7.2 ms, 9.3 ms, 11.4 ms, 13.5 ms, 15.6 ms, 17.7 ms, 19.8 ms, 21.9 ms, 24 ms] and [1.6 ms, 20 ms, 40 ms, 60 ms, 80 ms]. For both sections, the in-plane resolution was 4 mm by 4 mm, and the slice thickness was 4.2 mm. Diffusion-weighted MR imaging of the entire uterus was acquired with a 2D EPI sequence in 86 different weightings, with a max b-factor value equal to 900 s/mm^2^. Other acquisition parameters were: 12.8 s repetition time, 62 ms echo time, the in-plane resolution was 3 mm by 3 mm, and slice thickness was 3 mm. The total scan time was approximately 22 minutes.

**Pre-processing**

Both of the T2* scans are done by a relatively fast sequence which takes no more than 1min. The two sections of T2* were co-registered and merged. In Diffusion MR frames MP denoising and bias correction were conducted on each frame. The image registration in diffusion MR frames was done using Elastix. Elastix offered options in parameter tuning, a pre-optimized parameter set for 3D MR cervix T2 scan with non-rigid registration, and B-spline transformation^1^ downloaded from Elastix's official site parameter database. Additionally, the visual check was conducted after registration. Frames with unreasonable deformation or visually appreciable misalignment were excluded.

**T2* Computation**

The frames from two T2* MR acquisition sections were merged after co-registration by Elastix. A total of 16 frames were used to compute voxel-wise T2* time. The voxel-wise mono-exponential decay in T2* contrast follows equation 1:

|  | $S_{1}= S_{0}*exp(-\frac{t}{{T2}^{*}})$ | *Eq.1* |
| --- | --- | --- |

S1 was our voxel-wise measurement on T2* MR images, and t was the echo time defined in each multi-echo sequence. Two parameters estimated in T2* fitting for each voxel were ${T2}^{*}$ (the primary parameter) and $S_{0}$ (the initial signal intensity at t = 0 ms). T2* was the primary parameter in this study. Given previous studies and biological knowledge, all voxel-derived T2* values lower than 2 ms or higher than 150 ms were replaced with estimated values interpolated from the surrounding values. A customized MATLAB implementation of exponential curve fitting was used to fit two parameters simultaneously. A 3-D linear interpolation was applied on the T2* map to align it with diffusion MR images.

Elastix was used to register the placenta segmentations on T2* to those on diffusion b0 images with a rigid parameter set. The corresponding transform matrix was applied to the T2* volumes to achieve better alignment with diffusion tensor imaging volumes. The dice index between segmentation on the b0 image and the registered segmentation on the T2* image were computed to establish an exclusion criterion—cases with dice index < 0.95 were removed from the study to secure the data quality.

**Diffusion Tensor Imaging Computation**

Diffusion MR images with 86 different diffusion weightings were acquired. Due to fetal or maternal motion during the long scan period, image registration was performed to counter misalignment. Because registration did not fix every frame, a subset of well-aligned frames was identified by both visual check and reference to the motion coefficient. A subset including at least 25 diffusion-weighted frames and one non-diffusion-weighted frame was extracted as the input for the diffusion tensor imaging (DTI) model. Equation 2 was used to fit the diffusion tensor.

|  | $S_{1}= S_{0}* e^{-bD}$ | *Eq.2* |
| --- | --- | --- |

S1 and S0 were our measurements of the original signal intensity with and without, respectively, diffusion-weighting, b represented the intensity of diffusion weighting, and D was a 3 x 3 matrix that mathematically described the local diffusion tensor. DTI fitting was computed by an open-source MATLAB toolbox published by Vista Lab at Stanford University, Palo Alto, CA. Fractional anisotropy (FA), apparent diffusion coefficient (ADC), radial diffusivity (RD), and axial diffusivity (AD) were computed and used as input features for clustering.

**Appendix S2** Detailed description of the fuzzy clustering method based on the Gaussian mixture model and cross-validation

The multi-dimensional GMM is mathematically represented by equations 3 and 4.

|  | $p\left( \vec{x} \right)=\sum_{i=1}^{K} \varphi_{i}\mathcal{N}\left( \vec{x} \right\vert\vec{\mu_{i}} , \Sigma_{i})$ | *Eq.3* |
| --- | --- | --- |
|  | $\mathcal{N}\left( \vec{x} \vert\vec{\mu_{i}},\Sigma_{i} \right)=\frac{1}{\sqrt{{(2\pi)}^{K}\left\vert\Sigma_{i} \right\vert}}\exp\left( -\frac{1}{2}\left( \vec{x}-\vec{\mu_{i}} \right)^{T}{\Sigma_{i}}^{-1}(\vec{x}-\vec{\mu_{i}}) \right) , \sum_{i=1}^{K} \varphi_{i}=1$ | *Eq.4* |

K is the total number of underlying subpopulations and requires manual input or proper estimation. The data point $\vec{x}$is a vector element, which is the same as model dimensionality. The $K^{th}$ component has a mean of $\mu_{k}$ and covariance matrix of $\Sigma_{k}$. $\varphi_{k}$ is the weighting parameter in $K^{th}$ component and sums to one so that the joint probability distribution $p(\vec{x})$ is normalized. Weighting parameter$\varphi_{k}$, with a mean value $\mu_{k}$ and covariance matrix $\Sigma_{k},$ was estimated by an expectation-maximization algorithm. K was determined by the Calinski-Harabasz ^2^ criterion and was equal to three in this study.

A randomly selected half of the total voxels were used to estimate the GMM parameters from five patients with uncomplicated pregnancies. This training dataset contained 42,000 voxels in total. After training, the posterior component assignment probability for the voxels in the remaining patients was computed by Bayes' theorem and estimated model parameters. The equation for the data point $\vec{x}$ belongs to component $C_{i}$ as in Equation 5.

|  | $p\left( C_{i} \vert\vec{x} \right)=\frac{\varphi_{i}\mathcal{N}\left( \vec{x} \right\vert\vec{\mu_{i}}, \Sigma_{i})}{\sum_{j=1}^{K} \varphi_{j}\mathcal{N}\left( \vec{x} \right\vert\vec{\mu_{j},} \Sigma_{j})}$ | *Eq.5* |
| --- | --- | --- |

Note that the posterior probability $p\left( C_{i} | \vec{x} \right)$ does not sum to one. The normalized posterior probability was regarded as the degree of membership for data point $\vec{x}$ to component $C_{i}$. The equation for the degree of membership $d\left( C_{i} | \vec{x} \right)$ is defined in equation 6.

|  | $d\left( C_{i} \vert\vec{x} \right)= \frac{p\left( C_{i} \vert\vec{x} \right)}{\sum_{i=1}^{K} p\left( C_{i} \vert\vec{x} \right)}$ | *Eq.6* |
| --- | --- | --- |
|  |  |  |

We repeated the training process using 10000 voxels from patients' placenta, and all of them showed similar mean values of detected distributions (**Figure.S1**). The complete results are also summarized in the **Table S1** below. The respective optimized mean apparent diffusion coefficient (ADC, mm^2^s^-1^), fractional anisotropy (FA, a.u.), axial diffusivity (AD, mm^2^s^-1^), radial diffusivity (RD, mm^2^s^-1^), and T2* (ms) for each cluster were: cluster 1) 1.95, 0.29, 2.56, 1.61, 83; cluster 2) 2.73, 0.61, 4.45, 1.85, 74; and cluster 3) 2.29, 0.34, 3.16, 1.86, 46.

**Figure S1** Repeated training process using different sample volume. (a) Mean values of randomly selected 10 000 voxels from 22 patients (repeated 10 times). (b) Mean values of randomly selected 10 000 voxels from six patients (repeated 10 times).


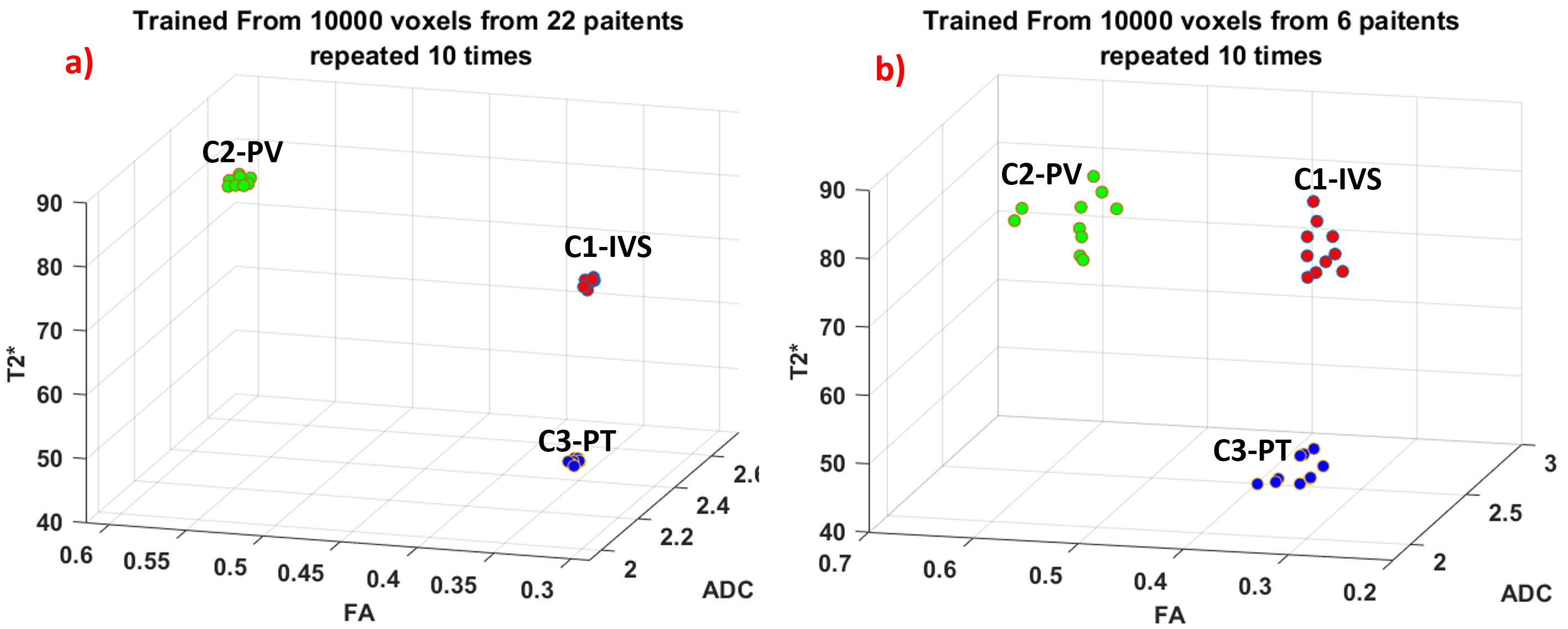


**Table S1** Repeated training process using different sample volume
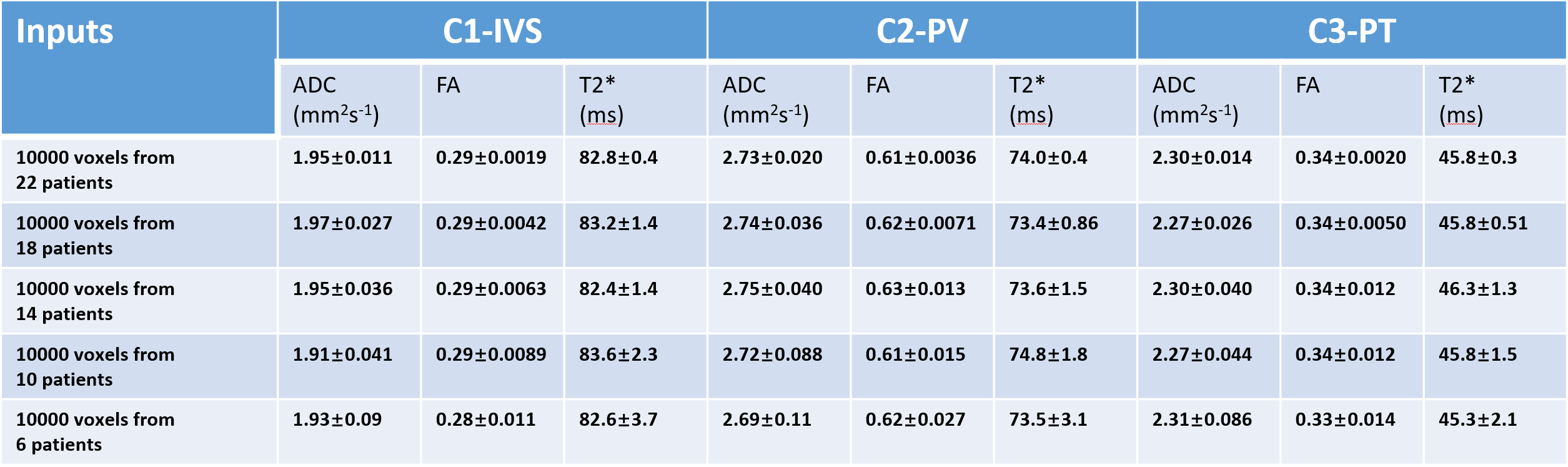


We also present the 3D presentation of 95% confidence ranges for the multi-variable gaussian distribution (FA, T2*, and ADC) as 3D shells and its 2D projections on three planes (**Figure S2**)

**Figure S2** (a–c) 95% CI of three-dimensional multivariable distribution. Red indicates intervillous space cluster, green indicates placental vessels cluster and blue indicates placental tissue cluster. (d–f) Planes showing the position of corresponding two-dimensional projections: 95% CI on apparent diffusion coefficient (ADC)-fractional anisotropy (FA) plane (d), 95% CI on ADC-T2* plane (e), 95% CI on FA-T2* plane (f).
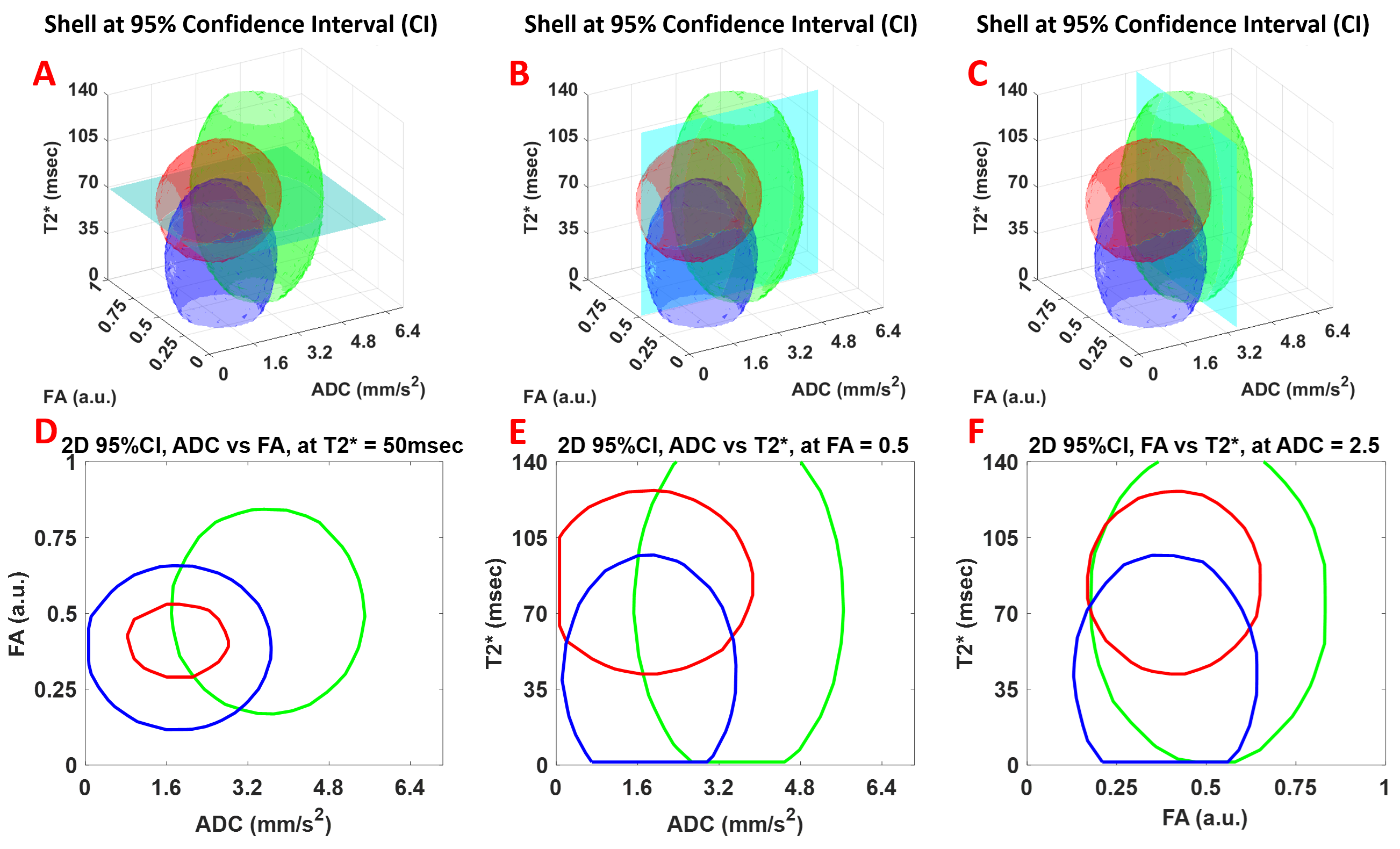


**Figure S3** Validation by manually selected voxels.


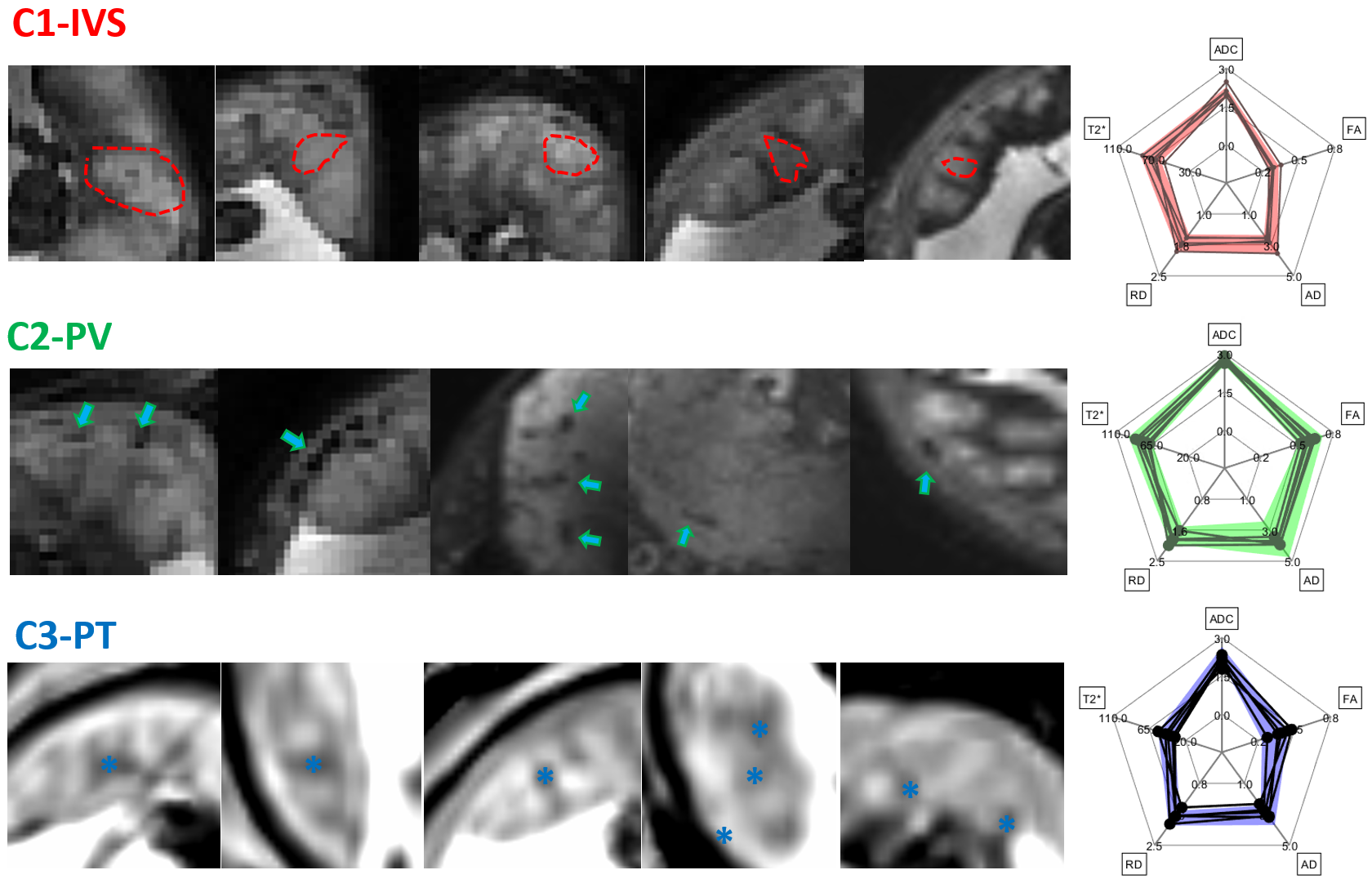


**Figure S3:** Experienced radiologists manually selected voxels in C1-IVS (red dash lines), C2-PV (green arrows), and C3-PT (blue arrows) from five healthy patients with confidence. C1-IVS and C2-PV were marked on DWI b0 images, C3-PT were marked on T2* map. For each of the five healthy patients, the average values of five features from all selected voxels were shown as solid dots and lines on the radar plot on the right. The color bands indicate the range of centroids from 22 healthy patients (data from **Fig1.F-H**). All manually selected placental voxels fell into the correct placental compartments, suggesting the accuracy of the proposed automatic placental segmentation methods.

**Figure S4** Flowchart summarizing selection of study subjects.

**
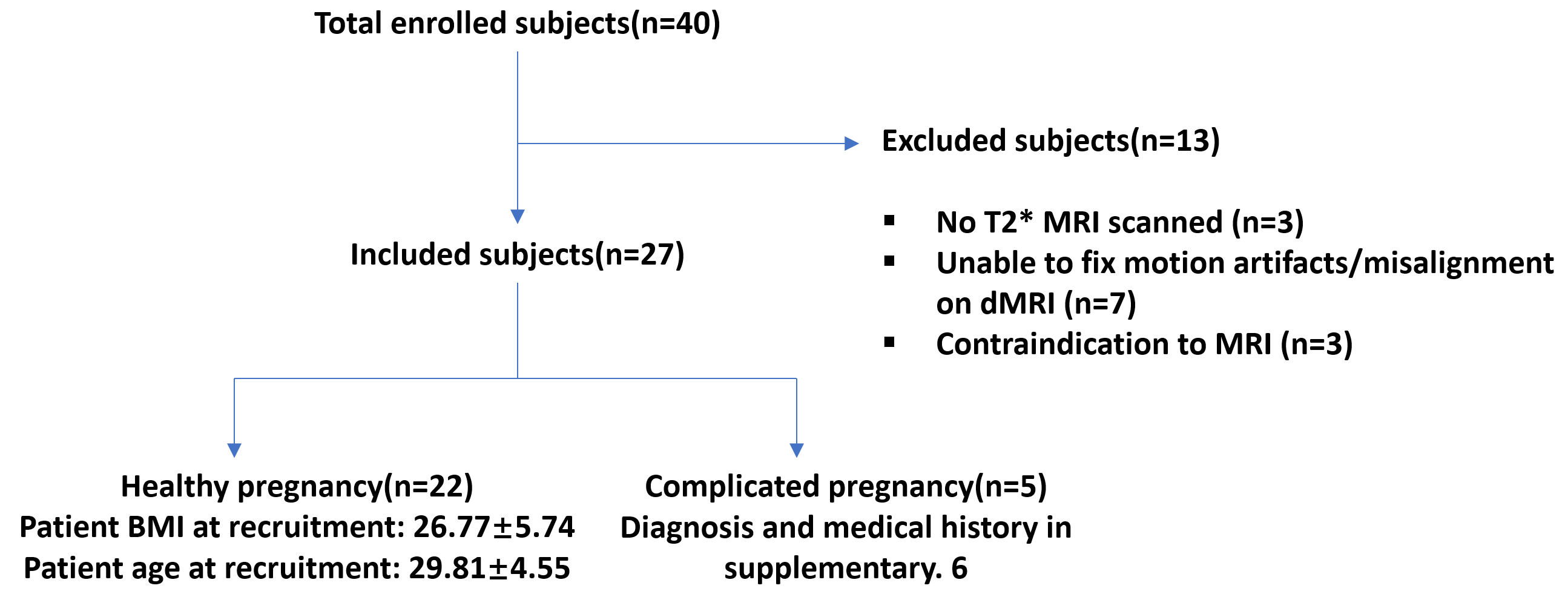
**

**Figure S5** Spatial distribution of placental compartments and oxygenation levels.

**
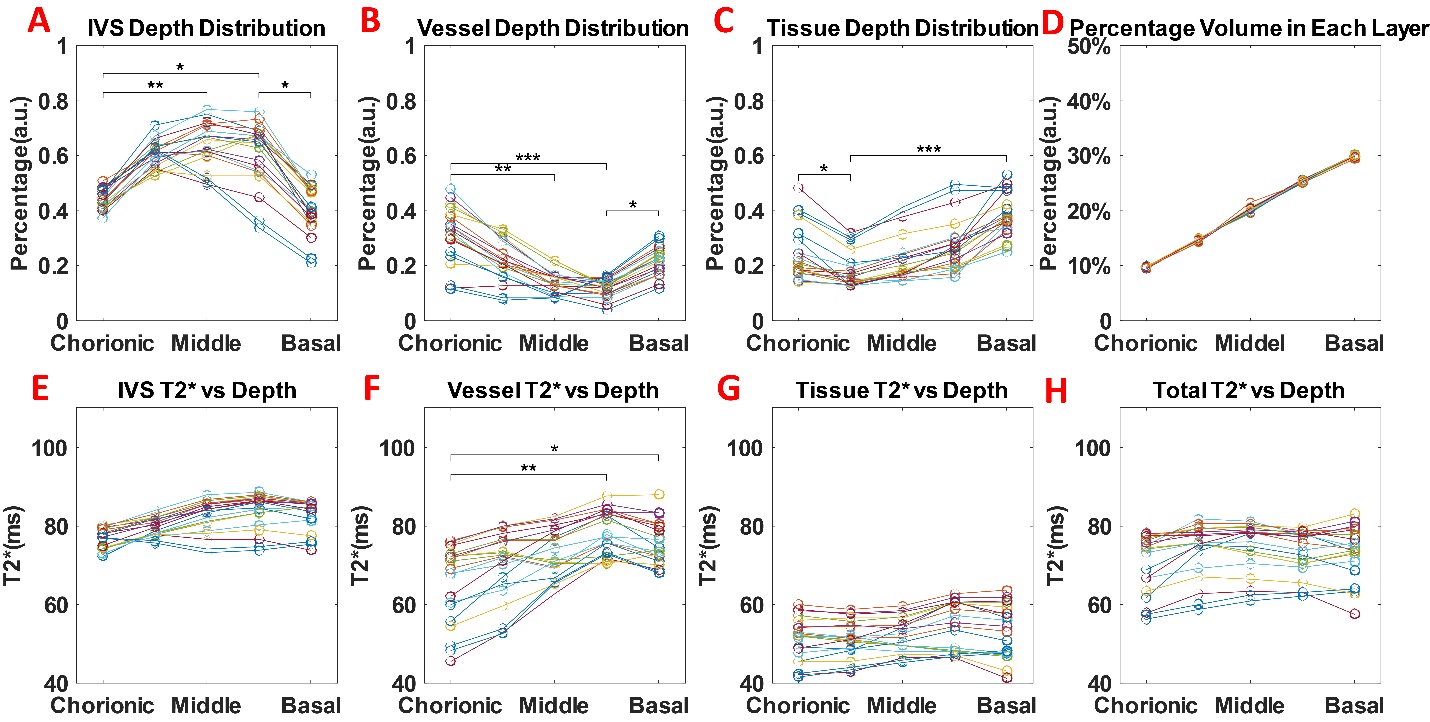
**

**Figure S5: Spatial Distribution of Placental Compartments and Oxygenation Levels**. A-C): The percentage existence of three clusters across five layers in all patients was presented. D): The total volume in each layer. Due to the curved shape of the in-vivo placenta. E-H): The depth distribution of T2* in the vessel, IVS, depth, and in total. Statistical exams were applied across layers. (p was computed by student two-sample t-test, symbol * indicating range of p-value: *p<0.05, **: p<0.005, ***: p<0.0005).

**Figure S6** Full spatiotemporal analysis.

**
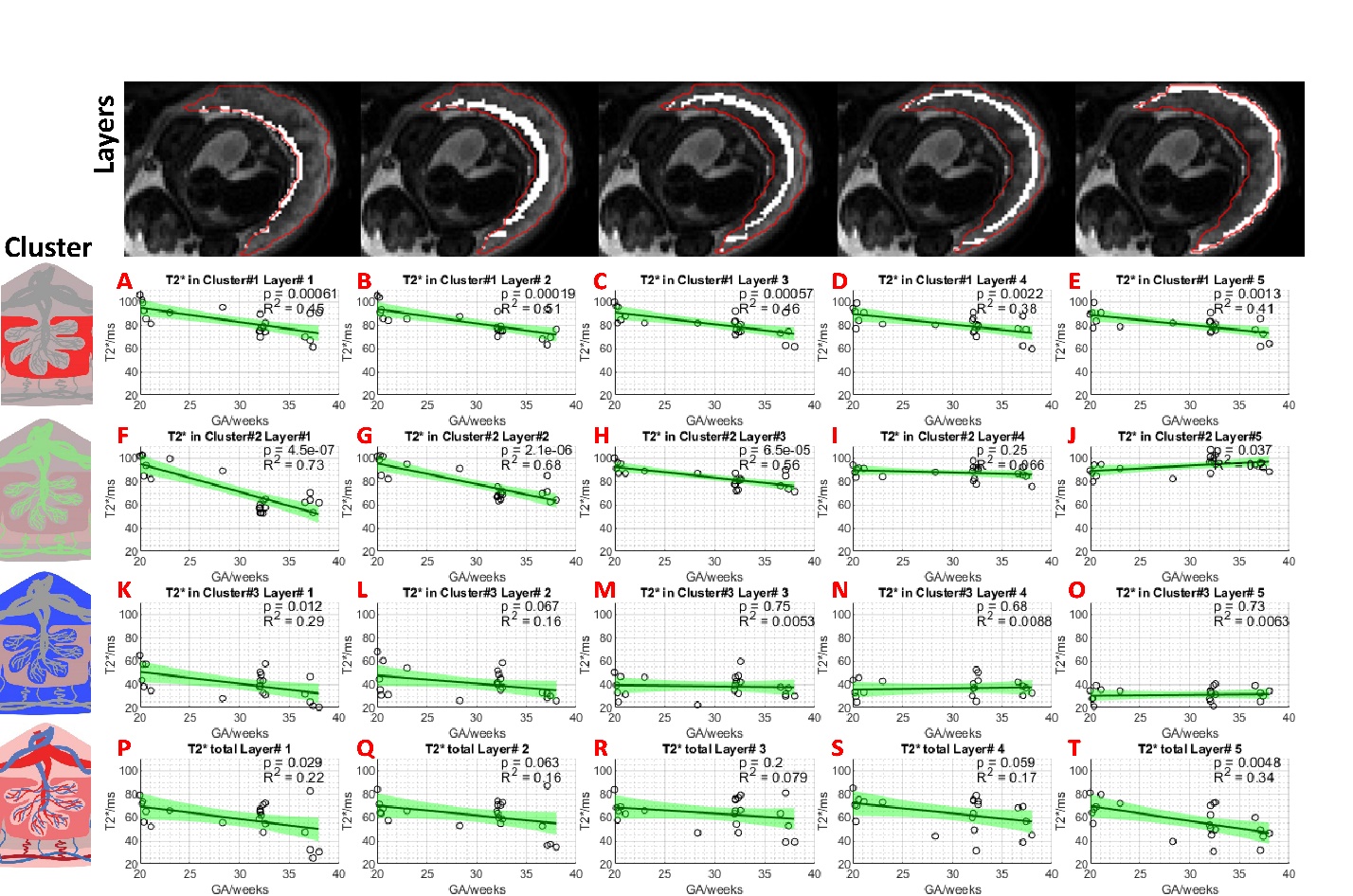
**

**Figure S6: Full Temporal-spatial Analysis.** Temporal-spatial analysis of T2* value in each cluster. A-E) longitudinal analysis of IVS cluster T2* value in layer#1 (close to chorionic plate) to layer #5 (close to basal plate). Each scatters in the plot represents a patient. Linear regression was applied in each plot with a light green band indicating a 95% confidence range. F-J) longitudinal analysis of vessel cluster T2* value across layers. With the panels arranged in the same order as A). K-O) longitudinal analysis of parenchyma cluster T2* value across layers. With the panels arranged in the same order as A). P-T): a longitudinal analysis of T2* value across layers but regardless of clusters.

**Appendix S3** Detailed description of the three identified clusters

Our data and findings suggest that cluster 1 represents the IVS for several reasons. First, voxels assigned to this cluster were predominantly in the middle of the placenta. Second, the voxels assigned to this cluster appeared to be divided by intervening C2-PV and C3-PT, which form placental functional units (cotyledons). However, the C1-IVS-assigned voxels are not completely separated from one another, as would be expected, given that the entire intervillous space is a connected volume filled with maternal blood. Third, voxels assigned to this cluster had low FA, as would be expected, given that maternal blood in an open space would have little restriction on free diffusion. Fourth, T2* values in this region were lower in the placentas of women at later gestational ages, reflecting increased fetal oxygen consumption as pregnancy progresses.

Furthermore, our findings suggest that cluster 2 represents the PV for several reasons. First, voxels assigned to this cluster were primarily localized to the chorionic and basal plates, where there are rich vascular structures. Second, voxels assigned to the C2-PV showed high FA values, as would be anticipated for the blood restricted to diffusing through blood vessels. Third, these voxels had high T2*, likely reflecting oxygenated hemoglobin in placental arteries, which we cannot differentiate from placental veins at the current imaging resolution. A single voxel (3 X 3 X 3 mm) could include multiple vessels with a width <1.2 mm. Fourth, as in the C1-IVS, T2* values in voxels assigned to C2-PV were lower in the placentas from women at later gestational ages. The increasing fetal metabolism results in increased deoxygenated hemoglobin in the blood. Finally, we found that T2* differences in C2-PV across gestational ages were the most pronounced on the fetal side of the placenta. This is consistent with the fact that the fetus consumes oxygen, resulting in more deoxyhemoglobin on the fetal side than on the maternal side.

Finally, the findings also suggest that cluster 3 represents the PT for two reasons. First, similar to the C2-PV, the C3-PT-assigned voxels are localized to the outer part of the placenta. Second, unlike in voxels assigned to C1-IVS and C1-PV, those in C3-PT did not have lower T2* values in the placentas of women at later gestational ages.
